# Ocular vs neurosyphilis. are they the same? A guide to investigation and management

**DOI:** 10.1038/s41433-024-03150-w

**Published:** 2024-06-24

**Authors:** Gerard A. Reid, Gabor Michael Halmagyi, Claudia Whyte, Peter J. McCluskey

**Affiliations:** 1 Ophthalmology Department, Belfast Health and Social Care Trust, Belfast, Northern Ireland UK; 2 Save Sight Institute, University of Sydney, Specialty of Ophthalmology, Faculty of Medicine and Health, Sydney, NSW Australia; 3 Department of Neurology, Royal Prince Alfred Hospital, Sydney, NSW Australia; 4 Central Clinical School, University of Sydney, Specialty of Neurology, Faculty of Medicine and Health, Sydney, NSW Australia; 5 Department of Infectious Diseases Prince of Wales Hospital, Sydney, NSW Australia; 6 Sydney Eye Hospital, Sydney, NSW Australia; 7 Department of Ophthalmology, Royal Prince Alfred Hospital, Sydney, NSW Australia

**Keywords:** Health care, Diseases, Signs and symptoms

## Abstract

This article reviews key concepts in the epidemiology, clinical features, diagnosis and management of ocular syphilis. It is not a systematic review or meta-analysis, but highlights the critical clinical features and investigations in patients with ocular syphilis. It reviews the overlap and interplay between ocular and neuro syphilis and provides practical guidance to diagnose and manage patients with ocular syphilis.

## Abstract (zh)

本文论述了眼部梅毒的流行病学、临床特征、诊断和治疗中的重要概念。本文并非系统综述或Meta分析, 但重点强调了眼部梅毒患者的重要临床特征和检查。文章回顾了眼部梅毒和神经梅毒之间的交叉和相互关系, 并为诊断和管理眼部梅毒患者提供了实用指导。

## Syphilis

**Fig. 1:**
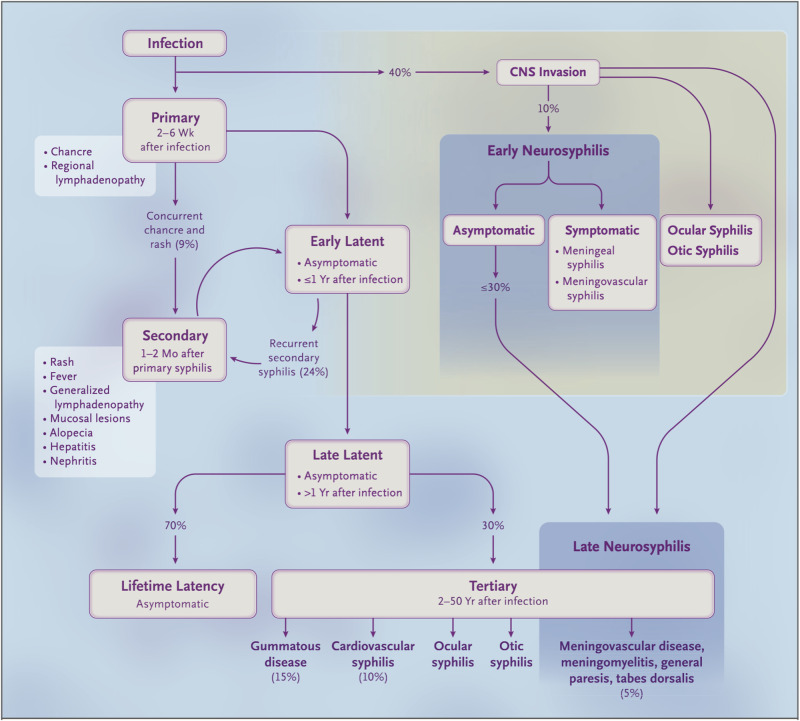
Natural history of syphilis infection from primary to tertiary stages including early CNS invasion (approx. 40% of cases) and both early and late neurosyphilis. Ocular syphilis, including optic neuritis can occur at any time point along this course. (Permissions from Ghanem, Ram, and Rice 2020, NEJM).

Syphilis is caused by infection with the obligate intracellular gram negative spirochaete, *Treponema pallidum*. Endemic treponemes such as yaws and pinta have been present in Europe and Asia for thousands of years however syphilitic infection has been recognised for only hundreds of years (from approx. the 15th century). The exact origins remain unclear, however, the “Colombian hypothesis” suggests syphilis was brought to the Western world by mariners returning from either Africa or the Americas and has subsequently spread throughout the world [1, 2]. Syphilis has been a disease of great interest since its recognition with a very large published literature. It is often described as “the great mimicker” as its clinical manifestations can imitate so many other diseases in many different organ systems. As Sir William Osler opined: “the physician who knows syphilis, knows medicine”. Ocular involvement has been recognised for many decades and occurs in different stages of syphilis (Fig. 1).

Syphilis is most commonly transmitted through unprotected sexual contact. The organism enters by crossing intact mucous membranes or via microabrasions in the skin. It may also be transmitted vertically from an infected mother to her unborn foetus, or very rarely by blood transfusion or other direct transmissions [3].

## Epidemiology

A peak of syphilis infection was recorded in the USA in the early 1940s when recording of infection rates was instituted. Subsequently there were many years of declining syphilis infection rates due to the availability of penicillin treatment. There was a second, but smaller, spike in infection rates in the 1980s and early 1990s during the HIV/AIDS pandemic. This was followed by another period of declining infection resulting from public health campaigns regarding safe sex habits [4].

However, since 2001, reported syphilis infection rates have increased every year in the USA, Europe, Australia and China [4–8]. There has been a corresponding rise in ocular and neurosyphilis presentations over this same time period [9–13]. Accurate estimation of syphilis prevalence remains problematic due to variable rates of reporting and are based on relatively small, published cohorts. Therefore, there is likely underestimation of syphilis prevalence.

Men who have sex with other men (MSM) are at increased risk of syphilis infection and account for the majority of all new male infections. Re-infection is not uncommon in MSM and should be considered in patients who re-present to the ophthalmology/neurology clinic with a history of having previously undergone treatment. Sex workers, illicit drug users and ethnic minorities are also at greater risk of syphilis infection [4–6].

HIV infection also increases the risk of syphilis infection with 7.1 infections in HIV positive MSM patients compared to 4.8 infections per 100 people in HIV negative MSM patients [6]. Factors contributing to increased risk in the MSM group are; an increase in high-risk sexual behaviour (i.e. unprotected sex, anal sex, group sex and drug use at the time of sex), serosorting (i.e. selecting partners based on HIV status) and a change in attitude toward risk and unprotected sex with the availability of HIV pre-exposure prophylaxis (PrEP) schemes [14, 15].

## Ocular syphilis

Ocular syphilis may involve any ocular tissue at any timepoint in the clinical course of syphilis infection. There is a wide range of ocular clinical phenotypes including interstitial keratitis, scleritis, orbital inflammation and neuro-ophthalmic presentations. Uveitis and optic neuropathies are the commonest ocular manifestations.

Papillitis was reported as the most prevalent clinical feature of uveitic syphilis over a 12 year period in a study from Spain [16] and a systematic review and meta-analysis of syphilitic uveitis identified the optic disc as the most frequently affected site [17]. Another series from Australia identified optic neuritis clinically in 27% of ocular syphilis cases [18]. Optic disc involvement in syphilis may occur in isolation or be a prominent clinical sign in a patient with syphilitic posterior or pan uveitis.

## Uveitis

Uveitis is the major manifestation of ocular syphilis. As any clinical phenotype of uveitis can develop secondary to syphilis infection, clinical signs are of little diagnostic use in patients with syphilitic uveitis with the possible exception of placoid syphilis. Indeed, the SUN (Standardisation of Uveitis Nomenclature) classification system recommends that the diagnosis of syphilis be based on positive serological testing rather than clinical signs (Table 1). It is now a widely accepted practice to test all patients with uveitis for syphilis. SUN recommends using a reverse sequence syphilis screening algorithm from the Centre for Disease Control (CDC) in the USA which is being increasingly adopted in centres outside the USA (Table 2) [19].

**Table 1:** Classification Criteria for Syphilitic Uveitis, SUN working group 2021.

**Criteria**		
1. Uveitis with a compatible uveitic presentation, including		
	Anterior uveitis OR	
	Intermediate uveitis or anterior/intermediate uveitis OR	
	Posterior or panuveitis with one of the following presentations	
		Placoid inflammation of the retinal pigment epithelium or
		Multifocal inflammation of the retina/retinal pigment epithelium or
		Necrotising retinitis or
		Retinal vasculitis
AND		
2. Evidence of infection with *Treponema pallidum*, either		
	Positive treponemal test and non-treponemal test	
	Positive treponemal test with two different treponemal tests	
**Exclusions**		
History of adequate treatment for syphilitic uveitis*		

*See also: Reverse Sequence Syphilis Screening Algorithm.

**Table 2:** Reverse sequence syphilis screening algorithm, Centre for Disease Control (USA).

Reverse Sequence Syphilis Screening Algorithm
1. Screen with a treponemal test, either an enzyme immunoassay (e.g. syphilis IgG) or a chemoluminescence assay (e.g. FTA*)
a. If treponemal test negative, syphilis is not present
b. If treponemal test positive, perform a non-treponemal test (e.g. RPR^†^ or VDRL^‡^)
2. Confirmation with non-treponemal test result
a. If non-treponemal test positive, patient has syphilis
b. If non-treponemal test negative, perform a different specific test (e.g. TP-PA^§^)
3. Second (different) treponemal test result
a. If second treponemal test result positive, patient has syphilis
b. If second treponemal test result negative, patient does not have syphilis (may have false positive test or treated syphilis)

*FTA fluorescent treponemal antibody. ^†^*RPR* rapid plasmin reagin. ^‡^*VDRL* Venereal Disease Research Laboratory. ^§^*TP-PA* Treponemal pallidum particle agglutination.

Adapted from:

Centres for Disease Control and Prevention. Syphilis. 2015 Sexually Transmitted Diseases Treatment Guidelines. Accessed 11 April 2019 at cdc.gov.

Centres for Disease Control and Prevention. Discordant results from reverse syphilis screening – five laboratories, United States, 2006–10. MMWR Morb Mortal Wkly Rep 2011;60:133–7.

**Fig. 2:**
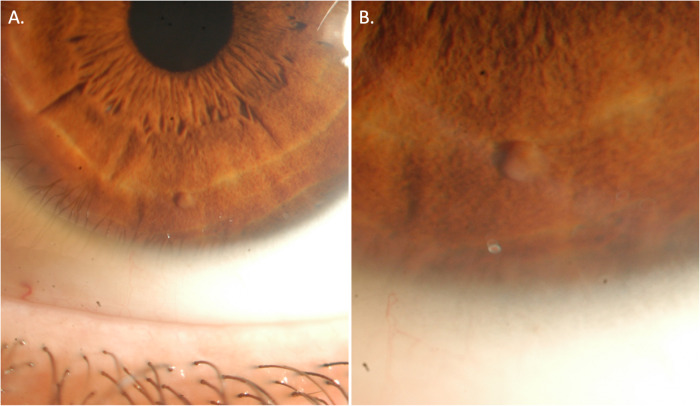
Anterior segment photographs of a patient with panuveitis and a diagnosis of ocular syphilis with concurrent HIV infection. **A** Anterior segment photograph depicting faint keratic precipitates and an inferior iris nodule. **B** An enlarged image of the iris nodule, which subsequently resolved with oral steroid in combination with IV penicillin G. This anterior segment finding should raise clinical suspicion of syphilitic uveitis as well as other granulomatous uveitis aetiologies.

Syphilitic anterior uveitis may present with a broad range of clinical features. In a series of patients presenting with acute anterior uveitis, there were only two patients diagnosed with syphilis from 241 patients (0.8%) [20]. Anterior segment clinical signs which might heighten the suspicion of syphilis include hypertensive anterior uveitis, the presence of iris nodules or of roseola (Fig. 2).

**Fig. 3:**
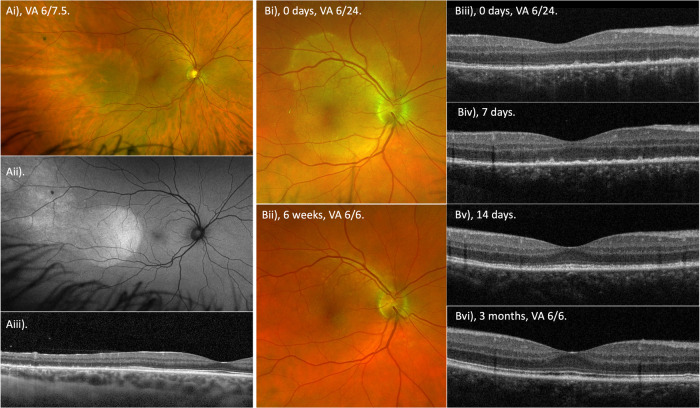
A sequence of fundus and OCT images, illustrating acute syphilitic posterior placoid chorioretinitis (ASPPC). **A** An ASPPC lesion, just temporal of the fovea in a patient with 6/6 vision but a symptomatic with a nasal scotoma. The placoid lesions is demonstrated on **Ai** widefield imaging, **Aii** widefield fundus autofluorescence present as hyperautofluorescence (note the temporal extension of the lesion) and **Aiii** Spectral domain OCT of the temporal macula illustrating normal outer retina bands of the ellipsoid zone (EZ), interdigitation zone (IDZ) and bruchs membrane/RPE complex (BM/RPE) at the central/nasal aspect of the macula. Temporally on the B-scan the EZ and IDZ are lost and the BM/RPE band becomes blurred with hyperreflective deposits. The hyperautofluorescence of the lesion results from the RPE autofluroescent signal not being absorbed by photoreceetpers/photopigment in the regions were the EZ and IDZ are absent. **B** A further case of an ASPPC lesion viewed on widefield imaging: **Bi** at presentation and **Bii** at 6 weeks, with resolution of the lesion and VA improvement from 6/24 to 6/6. Spectral domain OCT illustrates **Biii** loss of the EZ, IDZ bands and a ruffled appearance with hyperreflective deposits at the level of BM/RPE (at presentation, VA 6/24). At 7 days, **Biv** the EZ, IDZ bands are still absent and the pyramidal deposits at the BM/RPE were more clearly defined. By 14 days, **Bv** the EZ, IDZ bands have begun to recover and the pyramidal deposits have disappeared. By final follow-up at 3 months, **Bvi** the macular OCT demonstrates a marked recovery with all retinal bands easily identified and VA 6/6. It is hypothesised that this sequence of fundal changes occurs due to inflammation at the level of the choriocapillaris and retinal pigment epithelium with subsequent loss of the outer retinal bands that demonstrate the ability to recover following adequate treatment with systemic antibiotics and usually concomitant steroid.

Posterior segment features may be more suggestive of syphilitic uveitis. Acute syphilitic posterior placoid chorioretinitis (ASPPC) is almost exclusively caused by syphilis infection. ASPPC was described by Gass in 1990 as “large, placoid, yellowish lesions with faded centres at the level of the pigment epithelium in the macula and juxtapapillary areas” [21]. The lesion causes painless vision loss and visual function recovers if treatment with antibiotics and systemic corticosteroids is not delayed [22]. Multimodal imaging of ASPPC has determined that the lesion is at the level of the RPE causing RPE disruption and loss of the photoreceptor outer segments, both of which recover with treatment (Fig. 3) [23, 24]. Recent advances in retinal imaging with widefield autofluorescence aids in identifying placoid lesions, which are readily apparent as hyperautofluorescent lesions (Fig. 3aii). It is likely that ASPPC is the result of treponemal infection of the choriocapillaris and/or RPE. Some authors have described improvement of ASPPC cases with corticosteroid therapy alone and postulate an immune mediated pathogenesis for the lesion [25–27].

**Fig. 4:**
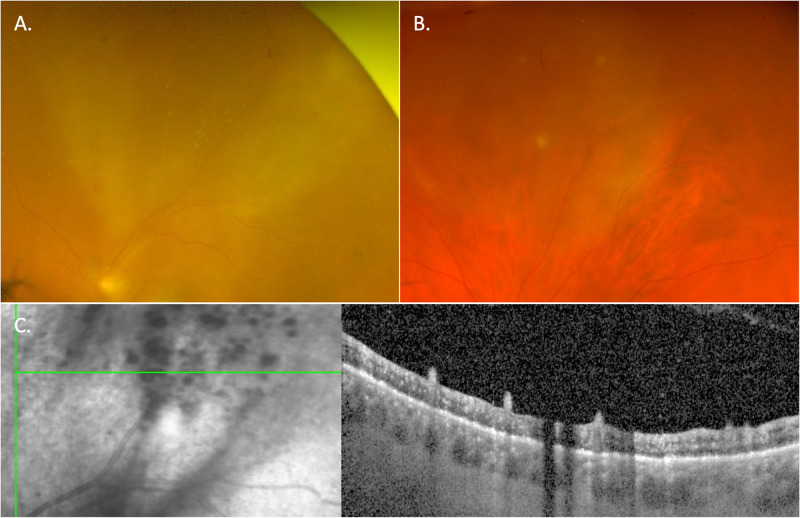
Punctate inner retinitis, with small, white pre-retinal precipitates. The precipitates measure from 50 to 500 μm and may occur in clusters. They are, however, always associated with underlying retinitis, sometimes described as ground glass appearance. **A** Widefield imaging of “ground glass” retinitis and pre-retinal precipitates, **B** the same lesion at 1 week during resolution of the lesion, and **C** spectral domain of the lesion demonstrates the pre-retinal precipitates and loss or thinning of the outer retinal bands associated with the underlying retinitis.

**Fig. 5:**
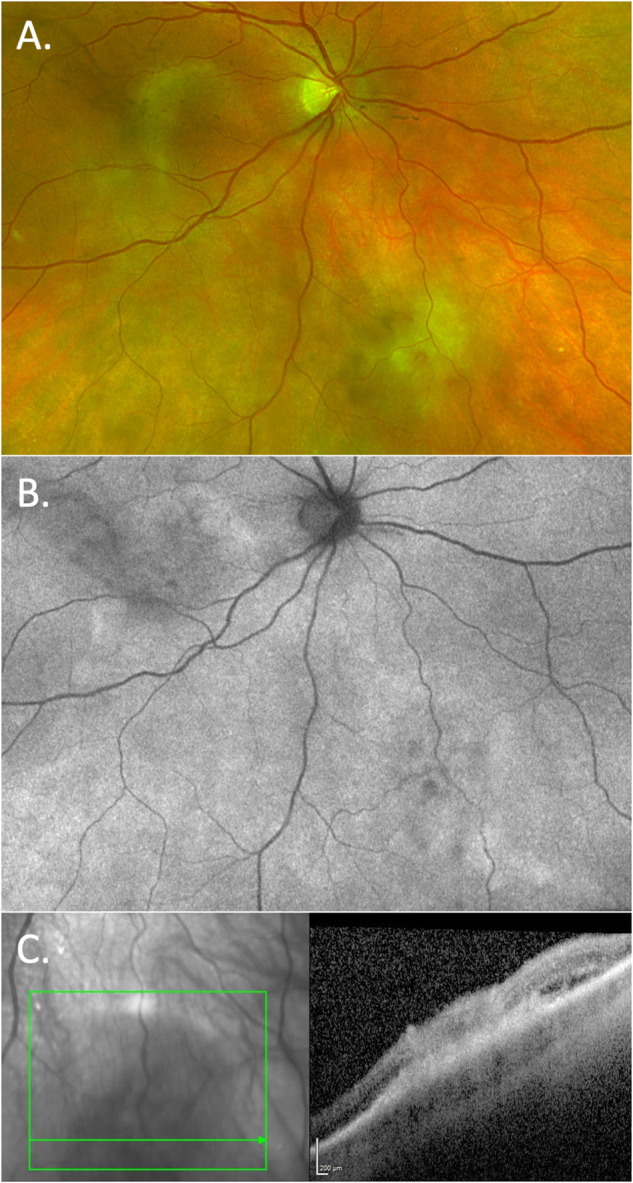
Focal retinitis in a patient with ocular and otic syphilis. **A** Fundal imaging of a macular placoid lesion and inferior area of focal retinitis. **B** FAF of the same area; hyperautofluorescence highlights the placoid lesion that extends inferiorly and incorporates the area of focal retinitis. **C** A spectral domain OCT of the focal retinitis demonstrating a full thickness retinal lesion, thickened RPE and preservation of the choroid.

**Fig. 6:**
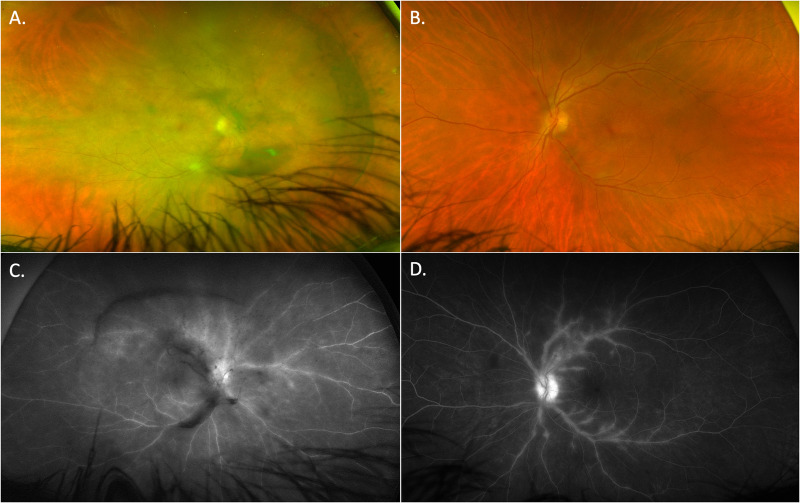
Bilateral panuveitis with vitritis and marked periphlebitis. Widefield imaging of the right (**A,** **C**) and left (**B,** **D**) eyes. **A,** **B** The perivascular sheathing is more apparent in the left eye with less vitritis, (**B)** widefield FAF, **C,** **D** clearly demonstrates the extent of peripheblitis in both eyes.

There are other recognisable clinical uveitis phenotypes where syphilis is an important differential diagnosis, including punctate inner retinitis with small, white pre-retinal precipitates which may be unilateral or bilateral (Fig. 4) [28, 29]. Necrotising syphilitic retinitis can closely mimic the peripheral retinitis of herpetic retinitis and acute retinal necrosis (Fig. 5). Finally, there can be prominent retinal vascular involvement presenting as retinal vascular occlusions, periphlebitis or arteritis (Fig. 6). Widefield fluorescein angiography has greatly enhanced our ability to recognise retinal vascular involvement.

HIV status does not appear have an negative impact on visual function following treatment of ocular syphilis but bilateral eye involvement may be more common and, depending on degree of immunosuppression, the degree of intraocular inflammation can vary [30].

## Neuroretinitis

**Fig. 7:**
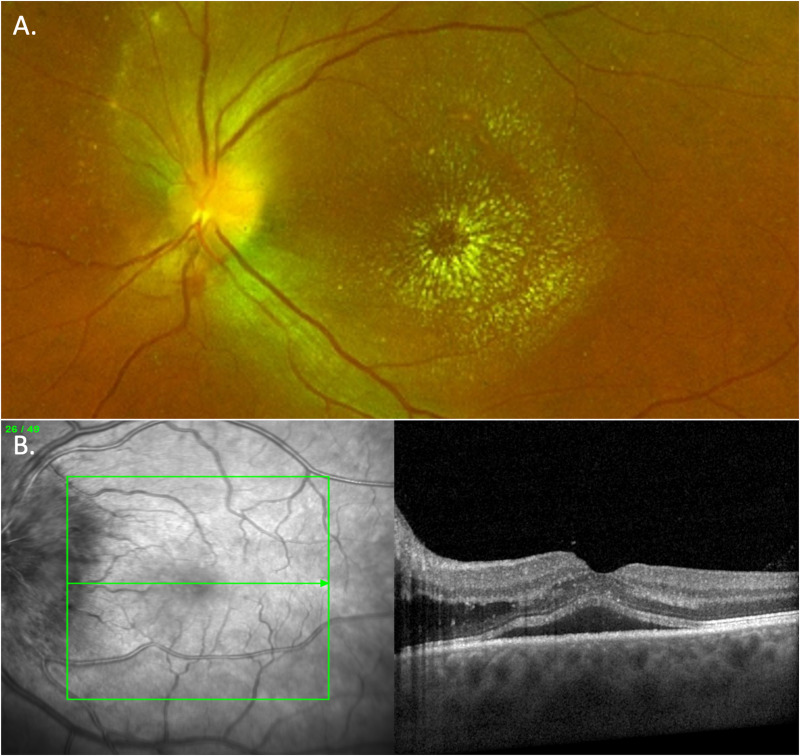
Neuroretinitis. **A** An example of neuroretinitis with macular star in a case of bartonella/cat scratch disease. **B** Spectral domain OCT from a case of ocular syphilis presenting with neuroretintis. Note the marked optic disc swelling, cystoid macular oedema nasal to fovea and subretinal fluid at the foveola.

Neuroretinitis is a rare ocular manifestation of syphilis. It is classically defined as a triad of subacute visual loss, optic disc swelling and a macular star (Fig. 7A) [31]. There may be overlying vitreous inflammation and there may be focal areas of retinal opacification in the epipapillary retina or posterior pole which on OCT involve the retinal nerve fibre and ganglion cell layer. Macular oedema and subretinal fluid are also frequent in this clinical presentation (Fig. 7B). It is most commonly unilateral.

## Optic neuritis and peri-neuritis

**Fig. 8:**
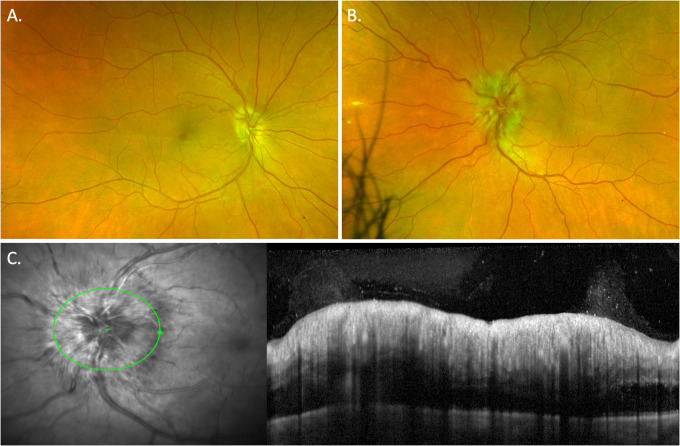
This patient presented with blurred vision in his left eye following a single dose of intramuscular Benzathine penicillin G. He had been treated by his general practitioner with a single dose of systemic antibiotic after incidental findings of positive result on syphilis serology. The single antibiotic dose undertreated treponema spirochaetes, presumably located at his optic nerve head and in the absence of systemic steroid lead to paradoxical worsening and development of an optic neuritis (**B)**, note that the right eye was unaffected (**A)**. Spectral domain OCT of the optic nerve **C** shows marked RNFL oedema/swelling and an associated vitritis.

Optic neuritis associated with syphilis may be unilateral or bilateral, follow an acute monophasic course, develop into a chronic progressive optic neuropathy or follow a relapsing remitting course. HIV infection seems to increase the likelihood of optic nerve involvement [30]. The optic disc may be clinically normal in retrobulbar neuritis and swollen in optic papillitis or anterior optic neuritis (Fig. 8). Optic atrophy may develop. As with other syphilitic ocular inflammatory manifestations it may mimic any other type of optic neuritis.

**Fig. 9:**
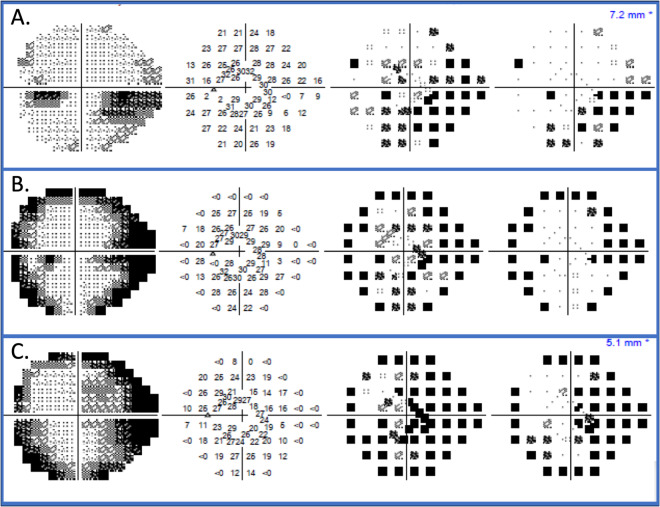
Serial 24-2 Humphrey visual fields in a patient with peri-neuritis. **A** The patient presented with mild VA reduction (6/9) and VF defect. There was a subsequent worsening of peripheral VF constriction over the follow-up period at **B** 1 week and **C** 2 weeks despite treatment with 14 days of IV penicillin G and IVMP pulsation followed by oral prednisolone.

Optic peri-neuritis is an orbital inflammatory or infective process that extends to involve the optic nerve sheath resulting in optic disc swelling and variable mild reduction in central vision, with constricted peripheral visual fields (Fig. 9). Orbital MRI demonstrates the nerve sheath enhancement and swelling.

## Neurosyphilis

Neurosyphilis describes a spectrum of neurological disorders, ranging from meningovascular involvement to late onset parenchymal disease such as general paresis, psychiatric disorders and tabes dorsalis. In the later stages of tertiary syphilis the infection is no longer contagious [32, 33]. There may also be prominent otic features of hearing loss and/or disturbance of vestibular function and ocular involvement at any stage of neurosyphilis.

It is difficult to define neurosyphilis and to identify markers of *Treponema pallidum* neuroinvasion. Clinical series have estimated neurosyphilis rates of between 0.47 to 3.1 cases per 100,000 population, an increase over recent decades [34–36]. The presence of neurological symptoms and signs with positive CSF abnormalities such as pleocytosis, elevated protein and/or a reactive CSF Venereal disease research laboratory (VDRL) test as well as peripheral blood treponemal antibody tests are used clinically to identify cases of neurosyphilis. These same CSF changes may be present in patients with ocular syphilis [37].

**Fig. 10:**
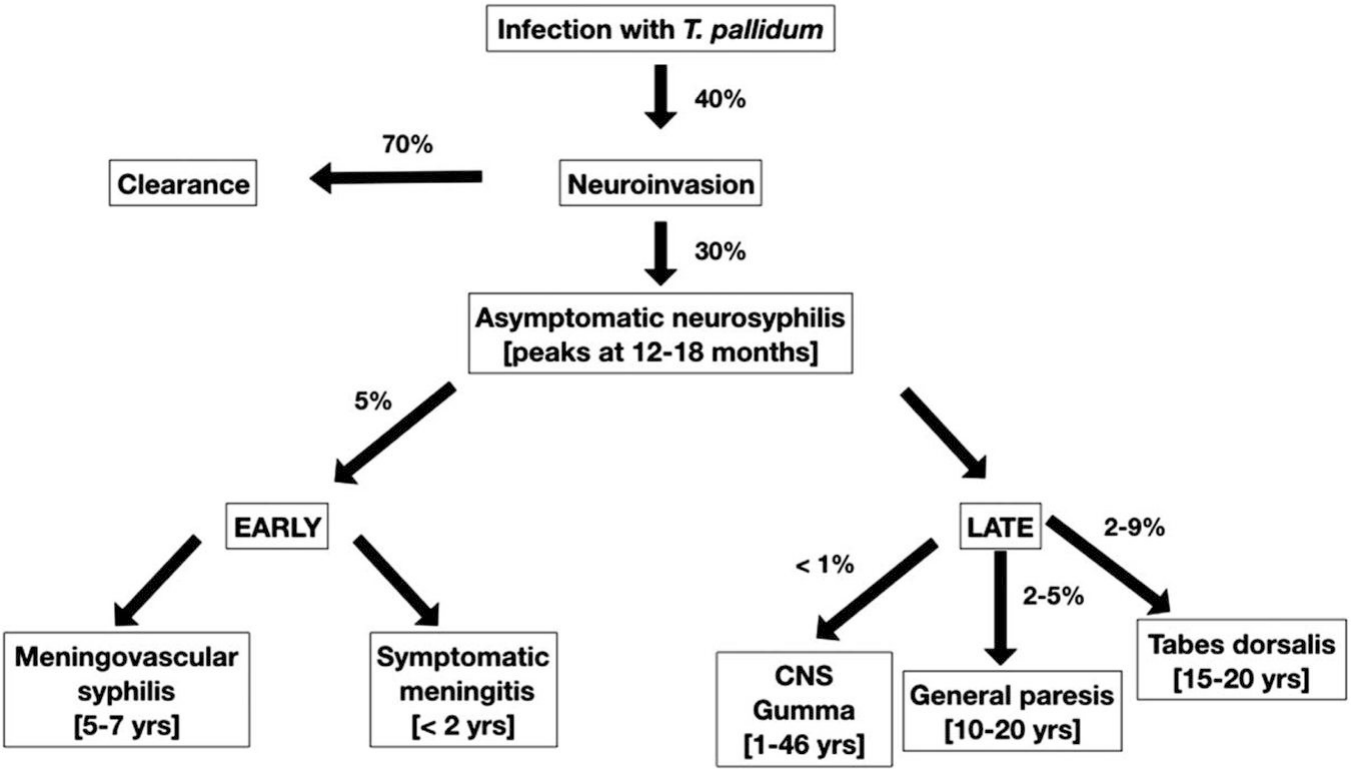
The natural history of neuroinvasion by *T. Pallidum a*nd the estimated progression to subsequent stages of neurosyphilis. (Permissions from A. Singh, Current Opinion in Infectious Diseases.2020).

Treponemal invasion of the central nervous system (CNS) is recognised to occur within days of primary infection. Lukehart et al. demonstrated that around 30% of patients with early syphilis (primary and secondary) had CSF evidence of syphilis neuroinvasion [38]. Subsequent studies report similar rates of 20-40% of syphilis infection with CSF abnormalities [33, 39, 40]. Many of these patients have early transient asymptomatic neurosyphilis that resolves without treatment. However, around 5% of these patients develop symptomatic acute syphilitic meningitis (Fig. 10) [41].

In later stages of untreated syphilis, CSF abnormalities are much less common [38]. A small number of infected patients develop late asymptomatic neurosyphilis that resolves, while an even smaller number of these patients go on to develop debilitating symptomatic late neurosyphilis. HIV co-infection does not increase the risk of neurosyphilis, but may accelerate the course of infection with the mean age of neurosyphilis diagnosis being younger in HIV infected patients, a finding not present in all forms of syphilis [10, 42, 43].

It may be that CNS spread of infection is a transient occurrence in many if not all syphilis infections with the vast majority so mild that they never meet the clinical threshold for CSF investigation and the infection is subsequently cleared from the CNS. That said, the presence of abnormal CSF results in the setting of syphilis does correlate with an increased risk of manifest neurological symptoms [39, 44]. There is still much regarding treponemal neuroinvasion that remains unclear.

## Symptomatic neurosyphilis

Early neurosyphilis is likely most prevalent as asymptomatic meningitis, and would only be diagnosed by CSF abnormalities. Symptomatic patients with early neurosyphilis present with headache and meningism. Further clinical manifestations at this stage are cranial nerve palsies (often multiple), ocular or otic involvement [33, 43].

Meningovascular syphilis is due to end arteritis of small and medium sized vessels and may cause ischaemic stroke or myelopathy. Meningovascular syphilis typically occurs 1 to 10 years after the primary infection [33].

**Fig. 11:**
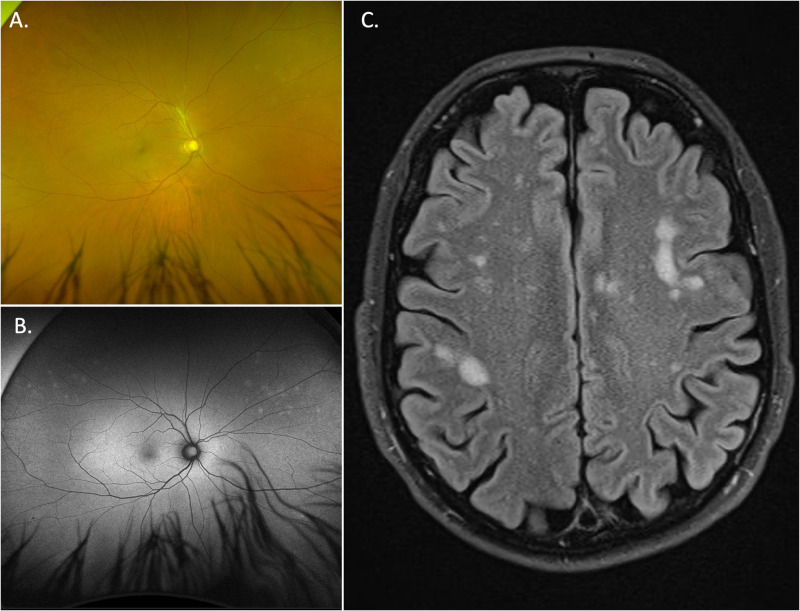
A case of neurosyphilis with ocular syphilis. The patient initially presented with blurred vision in his right eye, VA 6/15 and findings of anterior uveitis. **A** fundal examination demonstrated focal periphlebitis. **B** Widefield fundus autofluorescence reveals peripheral hyperautofluorescent flecks which are suspicious of syphilitic posterior uveitis and are easily missed on fundal examination alone. This patient was subsequently diagnosed with ocular syphilis and due to non-specific neurological symptoms had an MRI brain, **C** that revealed multiple, T2/FLAIR hyperintense sub cortical lesions.

Syphilitic gummas are rare and are granulomatous lesions more commonly seen in late neurosyphilis (but may arise in earlier stages). The neurological impact of gumma depend on their size and location. If a diagnosis of systemic syphilis infection has not been established and there is an absence of clinical suspicion, CNS lesions secondary to neurosyphilis may be mistaken for other common lesions such as inflammatory granulomas, metastases, lymphoma or meningioma (Fig. 11) [39].

Late symptomatic neurosyphilis, which develops decades after the primary infection, is now thankfully rare [45]. Generalised paresis (“general paresis of the insane”) progresses without treatment to a state of mental and physical collapse. Psychosis, depression, personality change, seizures and progressive dementia are prominent features. Tabes dorsalis is characterised by gait ataxia with Romberg’s sign and Charcot joints (neuropathic arthropathy). Argyll Robertson pupils (light-near dissociation) were a common sign in patients with late neurosyphilis [33, 46].

## Investigation

### Who needs a lumbar puncture?

Routine lumbar puncture is not necessary in patients with ocular syphilis as we treat all patients with posterior uveitis, pan uveitis and associated optic neuropathy as if they have neurosyphilis. This is based on evidence that approx. 60-70% of ocular syphilis cases have CSF abnormalities [47, 48].

It is not possible to identify patients at risk of developing symptomatic neurosyphilis and determining who needs investigations in the early phases of disease. At this time point, the diagnosis of neurosyphilis is based on a combination of clinical symptoms, clinical signs and abnormal CSF findings.

### Who needs a neurology or neuro-ophthalmology consultation?

Evaluation by a neurologist or neuro-ophthalmologist is important for ocular syphilis patients with neurological symptoms and for those with concurrent HIV infection. Those with hearing loss also need evaluation by an otologist [49]. Neurological evaluation could confirm a diagnosis of neurosyphilis and, more importantly, exclude other neurological disorders which may involve multiple anatomical sites and require alternative management. Ocular syphilis, neurosyphilis and otosyphilis patients all need lumbar puncture and CSF examination as part of their work-up and then careful follow-up to ensure adequate treatment for the best possible outcomes.

## Serological investigations

A diagnosis of syphilis relies on positive serology for syphilis and additionally, for a diagnosis of neurosyphilis, on CSF abnormalities including increased protein, pleocytosis and a positive CSF VDRL.

## Treponemal and non-treponemal testing

Specific tests react to treponemal antibodies against spirochaetes and include; fluorescent treponemal-antibody absorption (FTA ABS), Treponema pallidum haemagglutination assay (TPHA), Treponema pallidum particle agglutination assay (TPPA), microhaemagglutination assay for antibodies to Treponema pallidum (MHA-TP), and Treponema pallidum enzyme immunoassay (TP-EIA). They remain positive for life following infection and remain positive after successful treatment. A positive result does not inform the recency or activity of infection. False-positive results can occur from other inflammatory diseases such as systemic lupus erythematosus or from other infections especially other spirocheteal infections such as yaws, bejel and pinta.

**Fig. 12:**
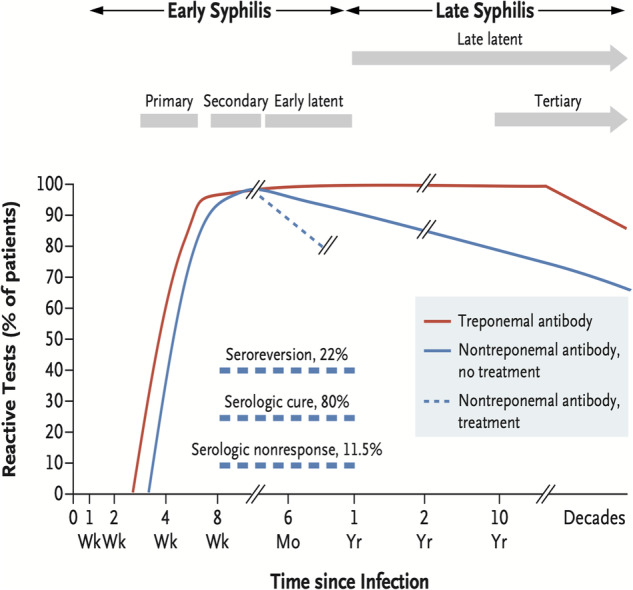
Graph depicting serological testing reactivity during the natural history of syphilis infection. Treponemal specific tests react to treponemal antibodies which once positive remain so for life (although there will be no reaction the first few weeks of primary infection). Non-treponemal testing react in response to cardiolipin-cholesterol lecithin antigen in active syphilis infection. With adequate treatment titres will normally return to a non-reactive level. Again, there may be no reactive result in early infection. (Permissions from Ghanem, Ram, and Rice 2020, NEJM).

Rapid plasma reagin (RPR) and VDRL are non-specific tests that detect cardiolipin antibodies produced in response to cardiolipin-cholesterol lecithin antigen in active syphilis infection. These tests are read by dilutional titration, decrease following treatment and increase with active infection. Usually a 4x fold titre decrease/increase is a significant result as a halving or doubling could result from reader interpretational error. With appropriate treatment, titres typically return to non-reactive or occasionally to a long term low titre (Fig. 12).

False negative results may occur with non-treponemal testing, especially in early primary/acute infection, and patients may need re-testing two weeks after initial presentation. False-positive non-treponemal testing can occur with infections such as tuberculosis, rickettsia and leprosy, in endocarditis and during pregnancy [33, 50, 51].

Availability of specific assays varies and it is critical that clinicians liaise with their local Infectious Disease physicians and laboratories to determine the available tests and their interpretation.

## Treatment

As with every aspect of syphilis there are no universally accepted guidelines for treatment. The CDC recommended treatment for isolated anterior uveitis in a patient with primary, secondary, and early latent syphilis is a single intramuscular (IM) injection of 2.4 million units (MU) of benzathine penicillin G. The CDC recommend treating patients with syphilitic posterior uveitis, panuveitis and optic nerve involvement, as neurosyphilis. The CDC recommended treatment for neurosyphilis is aqueous crystalline penicillin G (benzylpenicillin), 18–24 million units daily, given as 3–4 million units IV every 4 h or continuously infused, for 10–15days and most clinicians follow this guidance [52].

Penicillin allergy complicates management. Many patients who think they are penicillin allergic, when carefully questioned, are not. If there is doubt, skin testing can be performed. In selected patients desensitisation could be considered. Severe penicillin allergy necessitates using different antibiotics. Ceftriaxone 1–2 g intramuscularly (IM/IV) daily for 14 days has been documented as effective in treating patients with ocular syphilis. Doxycycline 100 mg twice per day for 28 days has also been used to successfully treat syphilitic uveitis [52, 53].

**Fig. 13:**
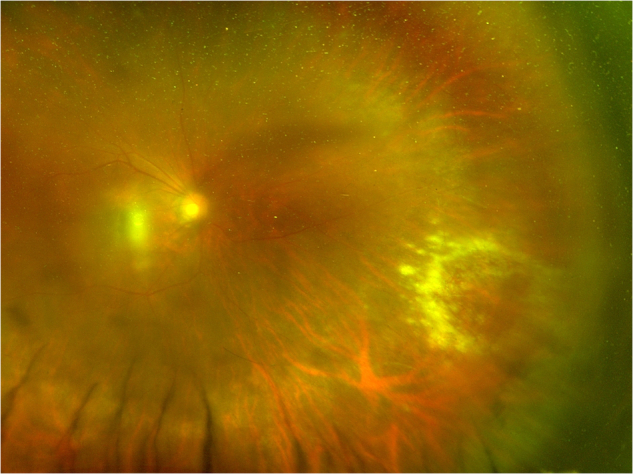
A case of CMV retinitis in the infero-temporal quadrant of an eye, we believe in response to local steroid (sub-tenons triamcinolone) as treatment for post operative (cataract surgery) CMO. Although these are rare complications, caution is required in patient with potential risk factors for such infection, e.g. HIV/immunosuppression.

Topical steroid therapy will control anterior segment inflammation in anterior uveitis. Systemic corticosteroid therapy is essential to limit the inflammatory mediated ocular damage in patients with posterior uveitis, panuveitis, and optic neuritis. A course of oral corticosteroids such as prednisolone beginning at 1 mg per kg and tapering over a minimum of 6-8 weeks is a widely used regimen and is standard practice in our unit. Pulsed intravenous methylprednisolone, peri-ocular or intravitreal corticosteroids have been reported to be effective in selected cases but should be used cautiously as the use of these medications has been correlated with poor outcomes, possibly due to their use in more severe ocular disease [54, 55]. Intravitreal dexamethasone or triamcinolone is effective therapy for treatment resistant macular oedema but consideration should be given to the possibility of activating a co-existing infection especially when there are additional risk factors. A classic example is reactivating CMV retinitis in HIV infected or severely immunocompromised patients following intravitreal triamcinolone (Fig. 13) [56].

Systemic corticosteroid therapy is required to control uveitis or optic neuritis in the setting of syphilis infection. As with other infectious forms of uveitis, paradoxical worsening can occur and cause significant vision threatening uveitis/optic neuritis if systemic corticosteroids are not used as part of the treatment regimen (as demonstrated in the case vignette of Fig. 8). The paradoxical worsening is analogous to that seen in patients with Tubercular uveitis and the immune reconstitution syndrome seen in HIV patients. We recommend starting oral prednisolone concurrently with antimicrobial therapy. Increased doses of corticosteroids (ie. greater than prednisolone 1 mg/kg) may be needed to control paradoxical worsening. We find that the Jarisch-Herxheimer hypersensitivity response that some patients develop secondary to spirochaetes lysed by antibiotic therapy is well controlled by the corticosteroid dose required to control syphilitic uveitis.

There are several important practical management points to emphasise.

1. The Ophthalmologist has a major role in the diagnosis and management of syphilis. Careful clinical assessment with a thorough history and ocular examination combined with a directed review of systems to detect risk factors for infection. If we don’t test for syphilis, we won’t diagnose syphilis. In patients with uveitis and optic neuritis, there is very rarely a good reason **not** to test for syphilis.
2. Multimodal imaging has greatly increased our diagnostic acuity to recognise ocular syphilis involving the posterior segment and optic nerve. OCT imaging, widefield digital and autofluorescence imaging, and widefield fluorescein angiography have transformed diagnosis and follow up of many uveitis disorders. As an example, in patients with ocular syphilis, placoid fundus lesions are now readily diagnosed with widefield imaging and recognition of these lesions is enhanced with fundus autofluorescence and OCT imaging (Fig. 3). Multimodal imaging in patients with optic neuritis (or a diagnosis of intermediate uveitis) may reveal subtle retinal involvement that is not apparent on routine clinical examination (Fig. 10). Not uncommonly, widefield imaging systems such as Optos (Dunfermline, UK) will obtain good quality fundus images when clinically, we struggle to view the fundus due to media opacity/vitritis, small pupils/posterior synechiae or a severely photophobic patient (Fig. 14).

**Fig. 14:**
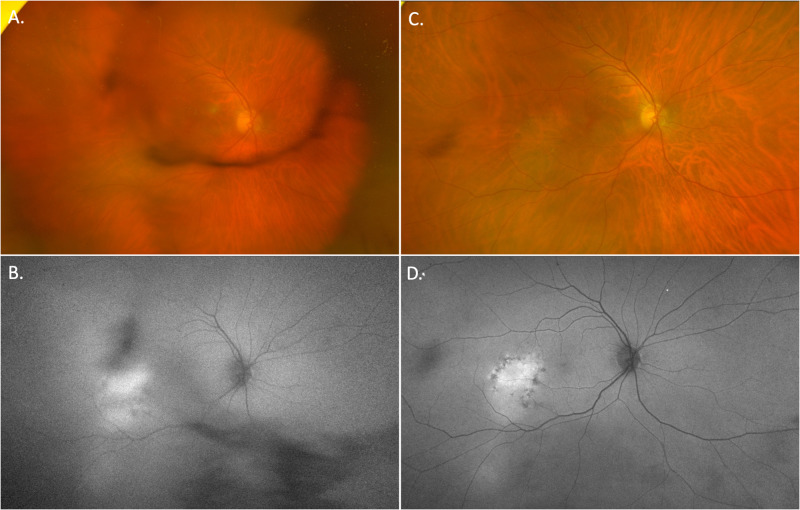
Widefield imaging of a case of ASPPC. The placoid lesion is obscured, **A**, due to mild vitritis and pigment on the anterior capsule from broken synechiae. Widefield FAF, **B** clearly identifies the despite the media opacity. Images **C**, **D** illustrate the placoid lesion, on widefield imaging and FAF respectively, days later after the media opacity had improved. We find SLO widefield imaging (e.g. Optos, Dunfermline. UK) will often identify posterior lesions obscured due to media opacity.
3. If at all possible, a multidisciplinary management approach is of great benefit to both the patient and the Ophthalmologist managing patients with ocular syphilis. This is standard practice in our unit. Sexual health or Infectious Disease physicians will assess for co-morbidities that might complicate treatment. Where syphilis is a notifiable disease, they will ensure the correct processes are followed and that contact tracing is performed. They will perform a sexual health screen and, critically, test for HIV infection.
4. Routine lumbar puncture is not necessary in patients with ocular syphilis as we treat all patients with posterior uveitis, pan uveitis and associated optic neuropathy as if they have neurosyphilis.
5. Patients with posterior or pan uveitis, or optic neuritis need 14 days of intravenous penicillin therapy which requires some form of central venous access (typically a PICC). In some locations, this will necessitate a 14 day hospital admission. In other centres where “hospital in the home” is available, many patients can be managed as outpatients. The medical team will follow the patient to ensure that syphilis has been effectively eradicated and manage patients with relapses or incomplete treatment responses.
6. In patients who have been adequately treated for ocular syphilis, a new presentation with uveitis could be due to a re-infection with syphilis. This is well recognised in the MSM risk group of patients.

## Issues requiring further research consideration

One of the uncertainties that remain for ophthalmologists and neuro-ophthalmologists managing patients with ocular syphilis is understanding the relationship between ocular and neurosyphilis and what the practical implications might be of further research in this area.

While we already know that most patients with syphilis get early spread of treponemes to the central nervous system, it appears that the majority do not develop clinical disease and instead clear the infection. A similar process may well be occurring within the eye, with early sub-clinical invasion and clearance and only a small number of patients having persisting intraocular infection. From a clinical perspective this is more of an academic interest, rather than related to direct patient care at this stage. However, it may give us insight into those who are at increased risk of future ocular and/or neurosyphilis. Understanding the risk factors and biomarkers of this risk, could allow better targeting of more aggressive treatment in the early phases of disease, as well as closer patient monitoring. This leads us back to the question of whether these biomarkers are likely to be found in the CSF, intraocular fluids or peripheral blood. There may also be genetically determined variations in innate immunity that alter susceptibility to ocular and neurosyphilis.

Looking at this question from the other side, there are likely to be patients with optic neuritis or symptomatic neurosyphilis with unrecognised ocular syphilis. Should all patients with optic neuritis to have multimodal imaging to look for retinal involvement, as this may affect longer term visual outcomes, or guide more localised treatment options? Should all neurosyphilis patients with or without visual symptoms have ocular multimodal imaging (widefield imaging including fundus autofluorescence) for the same reasons?

Finally, do we need to consider at what point ocular syphilis becomes neurosyphilis? Isolated anterior uveitis is an uncommon but important ocular manifestation of syphilis that usually occurs early in the natural history of syphilis and is adequately treated with a single dose of intramuscular penicillin. In contradiction, patients typically (but not always) develop posterior uveitis, panuveitis and optic neuritis later in the natural history of untreated syphilis and it is not uncommonly associated with asymptomatic or symptomatic neurosyphilis. For this reason, most centres including our unit treat all such patients with the treatment regimen used for neurosyphilis and follow up the patients for ocular relapse or development of other symptoms.

## Conclusion

This review has given an overview of the clinical features, diagnosis and management of ocular syphilis. It is not a detailed comprehensive review as multiple such reviews have been published over the past few years. This manuscript has focused on the practical diagnostic and management issues and addressed the relationship between ocular and neurosyphilis, which remains unclear despite many clinical cases series and reviews.
